# Complete Genome Sequence of a Listeria monocytogenes Strain from a Sample of Kale Salad

**DOI:** 10.1128/mra.00296-22

**Published:** 2022-06-28

**Authors:** Marc-Olivier Duceppe, Sohail Naushad, Amit Mathews, Mingsong Kang, Lin Ru Wang, Hongsheng Huang

**Affiliations:** a Ottawa Laboratory-Fallowfield, Canadian Food Inspection Agency, Ottawa, Ontario, Canada; b Greater Toronto Area Laboratory, Canadian Food Inspection Agency, Toronto, Ontario, Canada; University of Maryland School of Medicine

## Abstract

Listeria monocytogenes is a Gram-positive, rod-shaped, non-spore-forming bacterium that is an important foodborne bacterial pathogen for humans worldwide, with high mortality rates. Here, we report the complete genome sequence of a Listeria monocytogenes strain that was isolated from kale salad in Canada.

## ANNOUNCEMENT

Listeria monocytogenes, a pathogenic bacterium that can cause serious and sometimes fatal infections, is considered an increasing food safety concern worldwide (1). Here, we report the complete genome sequence of L. monocytogenes strain GTA-L356 (also referred to as CFIAFB20170086), which was isolated from a refrigerated closed bag of kale salad grown in Canada (purchased on 27 November 2017 and tested on 29 November 2017). Fifty grams of kale salad was introduced into 450 mL of UVM broth prewarmed at 30°C in a stomacher bag. Following 1 min of stomaching at speed 7 (Interscience BagMixer 400), the enrichment broth was incubated in the stomacher bag at 30°C for another 24 h. After this primary enrichment, 0.1 mL of the broth was transferred to 9.9 mL of morpholinepropanesulfonic acid-buffered *Listeria* enrichment broth (MOPS-BLEB) and further incubated at 35°C for 24 h. RLM and Oxford plates were streaked from the MOPS-BLEB, and colonies were confirmed using rapid Vitek (bioMérieux, Canada) identification (2). A single colony was isolated for further genomic DNA (gDNA) extraction.

gDNA was extracted from an overnight culture at 37°C in brain heart infusion medium using the Maxwell 16 cell DNA purification kit (Promega, Madison, WI, USA) for Illumina sequencing and the NanoBind CBB Big DNA kit (Circulomics, USA) for Nanopore sequencing. Different DNA preparations from different cultures were used for Illumina and Nanopore sequencing. The Illumina library was prepared using the Nextera XT library preparation kit (Illumina, USA) and sequenced for 600 cycles on an Illumina MiSeq system, which produced 936,395 paired-end reads. Nanopore sequencing was performed by MinION library generation using the 1D native (no shearing) barcoding gDNA protocol (EXP-NBD104 and SQK-LSK109; Oxford Nanopore Technologies, UK) and sequencing using a FLO-MIN106 (R9.4.1) flow cell and MinION MK1C system, which produced 64,208 reads (*N*_50_, 8.5 kb). Base calling was performed in super accuracy mode with Guppy v5.0.11. FastQC v0.11.9 (https://www.bioinformatics.babraham.ac.uk/projects/fastqc) and pycoQC v2.5.2 (https://github.com/a-slide/pycoQC) were used to assess read quality for Illumina and Nanopore reads, respectively. Long reads were trimmed and filtered with Porechop v0.2.3 (3) and Filtlong v0.2.1, respectively (4), and assembled with Flye v2.7 (5). The assembly was corrected using Medaka v1.4.4 (https://github.com/nanoporetech/medaka) and polished with short reads using a combination of NextPolish v1.4.0 (6), ntEdit v1.3.5 (7), and Polypolish v0.5.0 (8) after trimming/filtering with fastp v0.23.2 (9). Circlator v1.5.5 (10) was used to ensure that the assembly was circular and started with the *dnaA* gene. The depth of coverage was determined using minimap2 v2.17 (11) and SAMtools v1.13 (12) for long reads and BWA v0.7.17 (13) and SAMtools for short reads. Gene prediction and annotation were performed using the NCBI Prokaryotic Genome Annotation Pipeline (PGAP) v6.0 (14). Antimicrobial resistance (AMR) genes were identified using ResFinder v4.1.5 (15) and RGI v5.2.0 (16). Prophage sequences were analyzed using PHASTER (17). Parameters used for the genome assembly process can be found at https://github.com/OLF-Bioinformatics/nanopore/releases/tag/v0.1.

The complete genome of L. monocytogenes GTA-L356 contains a single chromosome of 2,957,755 bp and harbors a *fosX* AMR gene. Long- and short-read coverages were 267× and 136×, respectively. The annotated genome contains 2,843 proteins and has a GC content of 38.01%, similar to an average of 2,889 coding sequences (CDSs) and a GC content of 37.88% for L. monocytogenes genomes in the NCBI database (accessed 31 January 2022). The annotated genome contains 67 tRNAs, a 54.3-kb intact prophage (PHASTER score of 150) with 65 proteins and a GC content of 36.09%, and no plasmid.

### Data availability.

The complete genome sequence of L. monocytogenes strain GTA-L356 was deposited in GenBank (accession number CP092059). Base-called MinION and MiSeq reads in fastq format are available from the NCBI Sequence Read Archive (SRA) under accession numbers SRR17965222 and SRR17965216, respectively.

## References

[1] Radoshevich L, Cossart P. 2018. *Listeria monocytogenes*: towards a complete picture of its physiology and pathogenesis. Nat Rev Microbiol 16:32–46. doi:10.1038/nrmicro.2017.126. 29176582

[2] Pagotto F, Hebert K, Farber J. 2011. MFHPB-30: isolation of *Listeria monocytogenes* and other *Listeria* spp. from foods and environmental samples. *In* HPB methods for the microbiological analysis of foods, vol 2. Health Canada, Ottawa, Canada. https://www.canada.ca/en/health-canada/services/food-nutrition/research-programs-analytical-methods/analytical-methods/compendium-methods/methods-microbiological-analysis-foods-compendium-analytical-methods.html.

[3] Wick RR, Judd LM, Gorrie CL, Holt KE. 2017. Completing bacterial genome assemblies with multiplex MinION sequencing. Microb Genom 3:e000132. doi:10.1099/mgen.0.000132. 29177090 PMC5695209

[4] Kang M, Chmara J, Duceppe MO, Phipps-Todd B, Huang H. 2020. Complete genome sequence of a Canadian *Klebsiella michiganensis* strain, obtained using Oxford Nanopore Technologies sequencing. Microbiol Resour Announc 9:e00960-20. doi:10.1128/MRA.00960-20. 33184156 PMC7660995

[5] Kolmogorov M, Yuan J, Lin Y, Pevzner PA. 2019. Assembly of long, error-prone reads using repeat graphs. Nat Biotechnol 37:540–546. doi:10.1038/s41587-019-0072-8. 30936562

[6] Hu J, Fan J, Sun Z, Liu S. 2020. NextPolish: a fast and efficient genome polishing tool for long-read assembly. Bioinformatics 36:2253–2255. doi:10.1093/bioinformatics/btz891. 31778144

[7] Warren RL, Coombe L, Mohamadi H, Zhang J, Jaquish B, Isabel N, Jones SJM, Bousquet J, Bohlmann J, Birol I. 2019. ntEdit: scalable genome sequence polishing. Bioinformatics 35:4430–4432. doi:10.1093/bioinformatics/btz400. 31095290 PMC6821332

[8] Wick RR, Holt KE. 2022. Polypolish: short-read polishing of long-read bacterial genome assemblies. PLoS Comput Biol 18:e1009802. doi:10.1371/journal.pcbi.1009802. 35073327 PMC8812927

[9] Chen S, Zhou Y, Chen Y, Gu J. 2018. fastp: an ultra-fast all-in-one FASTQ preprocessor. Bioinformatics 34:i884–i890. doi:10.1093/bioinformatics/bty560. 30423086 PMC6129281

[10] Hunt M, Silva ND, Otto TD, Parkhill J, Keane JA, Harris SR. 2015. Circlator: automated circularization of genome assemblies using long sequencing reads. Genome Biol 16:294. doi:10.1186/s13059-015-0849-0. 26714481 PMC4699355

[11] Li H. 2018. Minimap2: pairwise alignment for nucleotide sequences. Bioinformatics 34:3094–3100. doi:10.1093/bioinformatics/bty191. 29750242 PMC6137996

[12] Li H, Handsaker B, Wysoker A, Fennell T, Ruan J, Homer N, Marth G, Abecasis G, Durbin R, 1000 Genome Project Data Processing Subgroup. 2009. The Sequence Alignment/Map format and SAMtools. Bioinformatics 25:2078–2079. doi:10.1093/bioinformatics/btp352. 19505943 PMC2723002

[13] Li H, Durbin R. 2010. Fast and accurate long-read alignment with Burrows-Wheeler transform. Bioinformatics 26:589–595. doi:10.1093/bioinformatics/btp698. 20080505 PMC2828108

[14] Tatusova T, DiCuccio M, Badretdin A, Chetvernin V, Nawrocki EP, Zaslavsky L, Lomsadze A, Pruitt KD, Borodovsky M, Ostell J. 2016. NCBI Prokaryotic Genome Annotation Pipeline. Nucleic Acids Res 44:6614–6624. doi:10.1093/nar/gkw569. 27342282 PMC5001611

[15] Bortolaia V, Kaas RS, Ruppe E, Roberts MC, Schwarz S, Cattoir V, Philippon A, Allesoe RL, Rebelo AR, Florensa AF, Fagelhauer L, Chakraborty T, Neumann B, Werner G, Bender JK, Stingl K, Nguyen M, Coppens J, Xavier BB, Malhotra-Kumar S, Westh H, Pinholt M, Anjum MF, Duggett NA, Kempf I, Nykäsenoja S, Olkkola S, Wieczorek K, Amaro A, Clemente L, Mossong J, Losch S, Ragimbeau C, Lund O, Aarestrup FM. 2020. ResFinder 4.0 for predictions of phenotypes from genotypes. J Antimicrob Chemother 75:3491–3500. doi:10.1093/jac/dkaa345. 32780112 PMC7662176

[16] Alcock BP, Raphenya AR, Lau TTY, Tsang KK, Bouchard M, Edalatmand A, Huynh W, Nguyen AV, Cheng AA, Liu S, Min SY, Miroshnichenko A, Tran HK, Werfalli RE, Nasir JA, Oloni M, Speicher DJ, Florescu A, Singh B, Faltyn M, Hernandez-Koutoucheva A, Sharma AN, Bordeleau E, Pawlowski AC, Zubyk HL, Dooley D, Griffiths E, Maguire F, Winsor GL, Beiko RG, Brinkman FSL, Hsiao WWL, Domselaar GV, McArthur AG. 2020. CARD 2020: antibiotic resistome surveillance with the Comprehensive Antibiotic Resistance Database. Nucleic Acids Res 48:D517–D525. doi:10.1093/nar/gkz935. 31665441 PMC7145624

[17] Arndt D, Grant JR, Marcu A, Sajed T, Pon A, Liang Y, Wishart DS. 2016. PHASTER: a better, faster version of the PHAST phage search tool. Nucleic Acids Res 44:W16–W21. doi:10.1093/nar/gkw387. 27141966 PMC4987931

